# A Recurrent Suprapituitary Ependymal Cyst Managed by Endoscopy-Assisted Transsphenoidal Surgery in a Canine: A Case Report

**DOI:** 10.3389/fvets.2019.00112

**Published:** 2019-04-16

**Authors:** László Lehner, Rita Garamvölgyi, Csaba Jakab, Zoltán Kerekes, Kálmán Czeibert

**Affiliations:** ^1^Felicavet Veterinary Clinic and Hospital, Budapest, Hungary; ^2^Medicopus Nonprofit, Ltd., “Kaposi Mór” Teaching Hospital of Somogy County, Kaposvár, Hungary; ^3^Private Veterinarian, Budapest, Hungary; ^4^VetScan Small Animal Diagnostic, Ltd., Budapest, Hungary; ^5^Department of Ethology, Institute of Biology, “Eötvös Loránd” University, Budapest, Hungary

**Keywords:** ependymal cyst, endoscopy, hypophysectomy, transsphenoidal surgery, canine

## Abstract

A 9-years-old spayed female mixed-breed dog was referred for the evaluation of intermittent head tremors, obtundation, long-standing blindness, and a tendency to seek confined spaces. The dog lost its vision 6 months before the current presentation. A menace response was absent on ophthalmological examination. Neurological examination did not show any abnormalities. A cyst measuring 16 × 18 × 14 mm was observed above the pituitary gland on magnetic resonance imaging. It extended toward the frontal area and compressed the optic chiasm and hypothalamic regions. A minimum preoperative database, including the findings of other required blood tests, was prepared. No abnormal laboratory findings were observed. Endoscopy-assisted transsphenoidal hypophysectomy was performed to remove the pituitary gland, drain the cyst, and partially excise the cyst wall. Normal pituitary gland tissue was observed on histopathology, and the mass was found to have a neuroendocrine or ependymal origin on cytology. Strict post-operative laboratory tests were performed at 1-h intervals for 24 h. An empty sella turcica region, and a collapsed and empty cyst wall was observed on follow-up magnetic resonance imaging. After 3 days of observation, the dog was discharged with a prescription of substitution therapy. However, the dog presented with the same signs and symptoms 73 days after the surgery. Cyst recurrence was apparent on magnetic resonance imaging. The owner requested euthanasia, and an ependymal cyst was observed on necropsy. To the best of our knowledge, we present the first case of an intra- and suprasellar ependymal cyst, and its surgical management in a canine. The findings from this case suggest that endoscopic transsphenoidal drainage and hypophysectomy could be a good surgical approach in cases where involvement of the pituitary gland is confirmed or strongly suspected on the basis of cytological and imaging findings.

## Background

Choroid plexus tumors account for 10% of all intracranial tumors in dogs, with the lateral, third, and fourth ventricles being the commonly involved sites (1). Based on this study the most affected breed is the Golden Retriever, and female predominance has been noted. Magnetic resonance imaging (MRI) shows hyperintense and hypointense areas on T2-weighted (T2W) and T1-weighted (T1W) imaging, respectively. A previous report described the use of suboccipital craniotomy for complete removal of a 5.5-mm choroid plexus cyst in a male Toy Fox Terrier (2). Another report described a cerebellar ependymal cyst (3) in a male Staffordshire Terrier. A quadrigeminal cyst (QC) is an intracranial intra-arachnoid cyst that develops because of arachnoid duplication. A previous study involving retrospective evaluation of 4,100 magnetic resonance images found 28 QCs affecting dogs (4). According to the findings, a hyperintense area in the midline, dorsal to the midbrain, caudal to the occipital lobe, and rostral to the cerebellum on T2W images is a characteristic MRI marker of QCs. Intracranial intra-arachnoid diverticula and cyst epithelial lining filled with fluid can cause compression and result in obstructive hydrocephalus. The fluid is cerebrospinal fluid, which can flow into the cyst either directly or via an osmotic gradient; MRI shows a hypointense area on T1W images, a hyperintense area on T2W images, and a non-contrast-enhancing lesion on fluid-attenuated inversion recovery images (5, 6). Most cases involve small-breed dogs, predominantly those with brachycephaly, and Shih Tzus tend to be overrepresented. In a study describing the MRI findings of QCs in five dogs, the cyst was located in the dorsal-median area of the cerebellum (7). The MRI findings and surgical management of a neuroendodermal cyst have also been described (8). Other reports involve a brainstem arachnoid cyst in the pontomedullary region that caused unilateral facial paresis in an 8-years-old female Maltese dog (9) and a caudal fossa respiratory epithelial cyst in the fourth ventricle that caused bilateral vestibular syndrome in an 11-months-old female Bloodhound (10). Meningioma is also a common intracranial tumor in dogs, although cystic meningioma is rare and has been described in only one study involving three dogs (11). Epidermoid cysts are true and rare cysts involving the central nervous system of dogs. These cysts are thought to be derived from aberrant inclusion of the non-neural ectoderm in or on the neural tube during embryogenesis. The predilection site is the caudal fossa, and vestibular and cerebellar signs can be observed (12). The MRI and histopathological findings of these cysts have been described in previous studies (12–15). One study also described the surgical removal of an epidermoid cyst in the fourth ventricle via suboccipital craniotomy and partial first cervical laminectomy (16). Cell proliferations of the pituitary gland are common in dogs, and result in the formation of micro- or macroadenomas, adenocarcinomas, hyperplasia, craniopharyngeal duct cysts, secondary neoplasms, and hypophysitis (17). Functional disturbance of the pituitary gland, which causes canine hypercortisolism (central Cushing syndrome), is found in most cases (18, 19). Microadenomas measure <10 mm while macroadenomas exceed 10 mm in size (18–20). Many studies have recommended preoperative radiation therapy for macroadenomas (21, 22). Since 1928, several surgical techniques have been described in the literature. Transbuccal (23), transcranial (24), and transsphenoidal (18, 25–27) techniques, with and without a neuronavigation system (28), have been used to approach the pituitary gland in dogs. Mamelak et al. used a high-definition video telescope for surgery using the pilot hole technique (29). Parasitic infections (toxoplasmosis) can also cause cyst-like lesions within the brain tissue of dogs (30). To the best of our knowledge, canine suprapituitary cysts and their surgical management have not been reported in the literature. Here we describe a recurrent suprapituitary ependymal cyst that was managed by endoscopic transsphenoidal surgery in a mixed-breed dog.

## Case Presentation

A 9-years-old spayed female mixed-breed dog was referred for the evaluation of moderate neurological signs. It tended to seek narrow places, experienced body tremors, and had lost its vision 6 months before the referral, although its eyesight had been weakening since 2 years. The owner was provided detailed information on the diagnostic and surgical procedures required, and consent was also obtained. A bilateral menace response was absent on neurological examination, with no other abnormalities. Normal findings were obtained on performing echocardiography and abdominal ultrasound. The left and right adrenal glands measured 56 and 57 mm in length, respectively. Minimal increase in alanine aminotransferase (312 IU/L; reference, 5–60), gamma glutamyltransferase (64 U/L; reference, >9 U/L), and lipase (521 U/L; reference, 24–108) levels, and a moderate increase in the alkaline phosphatase (973 U/L; reference, <280) level was observed on complete blood count, a chemistry panel, and urinalysis. The thyroxine (T4) level was slightly decreased (13.6 nmol/l; reference, 17–54). MRI and computed tomography were recommended for further assessments.

Following intravenous cannulation, the dog was anesthetized using propofol injection (5 mg/kg body weight [bwkg]; Narcofol®, CP-Pharma GmbH, Burgdorf, Germany). After intubation, anesthesia was maintained with a mixture of isoflurane and oxygen gas (Forene®, AbbVie Deutschland GmbH & Co. KG, Wiesbaden, Germany; 1.5% volume/volume; oxygen flow, 2 1/min). MRI was performed using a 1.5-T device (Siemens Magnetom Avanto, Siemens, Erlangen, Germany) to acquire the following sequences: T2W images in the transverse (echo time [TE]/repetition time [TR], 112/4,220 ms; slice thickness [SL], 3 mm), sagittal (TE/TR, 112/3,500 ms; SL, 3 mm), and dorsal (TE/TR, 112/3,500 ms; SL, 3 mm) planes; thin-slice native images (TE/TR, 9.1/550 ms; SL, 2.5 mm); fat-suppressed images (TE/TR, 9.1/749 ms; SL, 0.9 mm); post-contrast images (0.2 mL/bwkg; Dotarem 0.05 mmol/l injection, Guerbet, Villepinte, France); T1W three-dimensional images (magnetization prepared rapid gradient-echo) in the sagittal, transverse, and dorsal planes; and time-of-flight angiography (TE/TR, 7/25 ms; SL, 0.7 mm). The field of view was 170 × 170 mm.

During the same session, native, and post-contrast computed tomography images of the brain were obtained using an inner ear (SL, 1.0 mm; kernel, H60s; SL, 0.75 mm; kernel, H70h) and carotid angio (SL, 2.0 mm; kernel, B30f; SL, 0.6 mm; kernel, B26f) protocol, followed by maximum intensity projection reconstructions in the dorsal plane.

**Figure 1:**
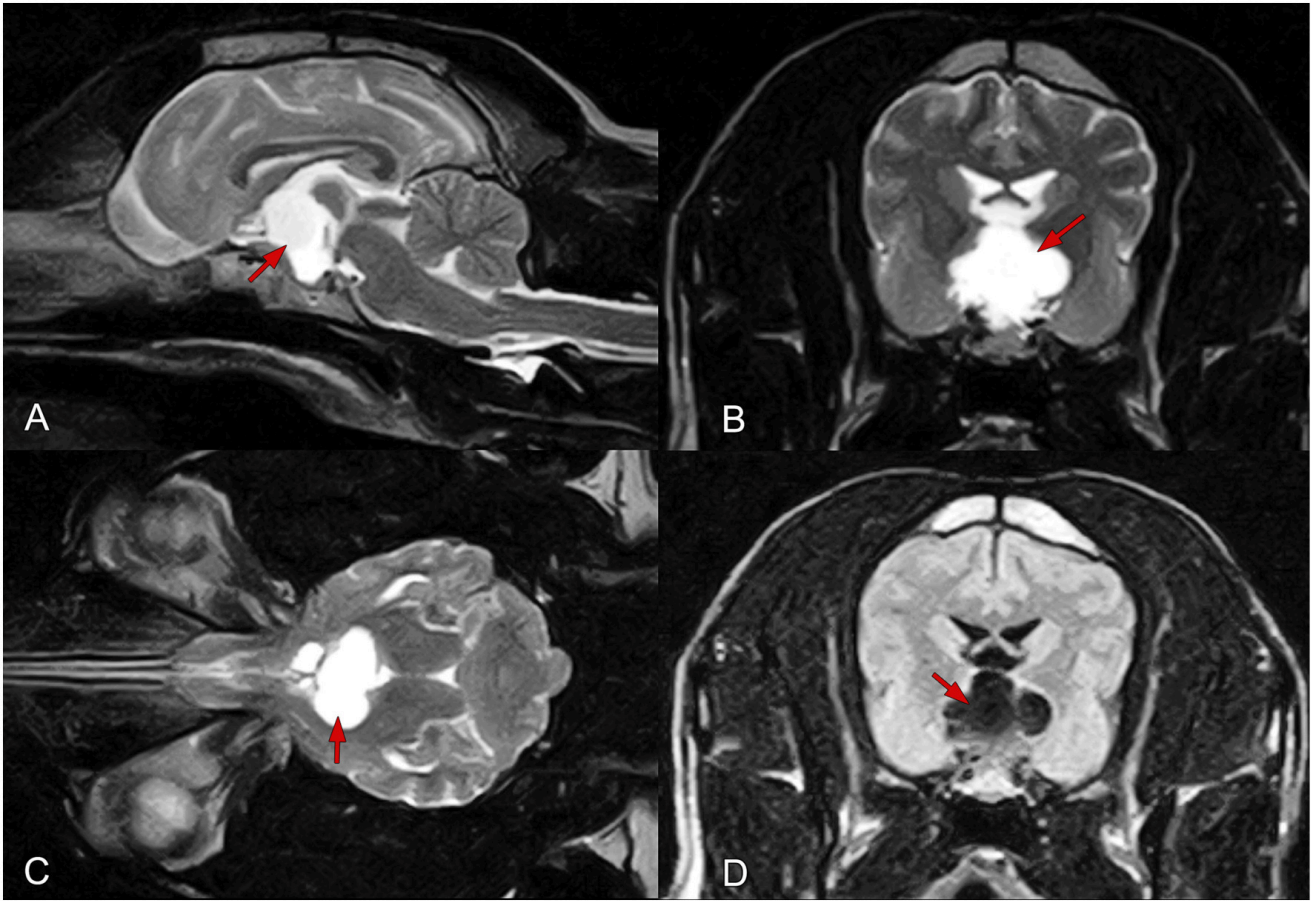
Findings of preoperative magnetic resonance imaging of the brain in a female mixed-breed dog with a suprapituitary ependymal cyst. **(A–C)** T2-weighted images in the sagittal **(A)**, transverse **(B)**, and dorsal **(C)** planes. **(D)** A fluid-attenuated inversion recovery image in the transverse plane. The red arrows show the fluid-filled cyst.

An intra- and suprasellar mass was observed on MRI (Figure 1). The intrasellar portion appeared iso- and hyperintense on T2W images, and hyper- and hypointense on T1W images, with marked contrast enhancement along the dura mater. A multicompartmental cyst-like component was attached to the mass; this component showed T1 hypointensity and T2 hyperintensity, and compressed the thalamus and pons in the caudal direction. The optic chiasma was also compressed in the cranioventral direction. The cystic structure showed late enhancement, particularly in the fluid-filled areas. The size of the cyst was 16 × 18 × 14 mm. The radiological diagnosis was an intra- and suprasellar lesion connected to a multicompartmental fluid field. Differential diagnoses included suprasellar arachnoid cyst, epidermoid cyst, craniopharyngioma of the pituitary gland, and myxomatous tumors.

Preoperative three-dimensional planning was performed using MeshMixer (AutoDesk, Inc., San Rafael, CA, USA) and Amira for LifeSciences 6.0 software (Thermo Scientific, Waltham, MA, USA) for identification of the landmarks for the surgical approach, and measurement of the exact volume and extension of the lesion. During surgery, the dog was placed in sternal recumbency, and its mouth was held open with a special equipment. Videoendoscopy was used for better visualization (Karl-Storz 2.7 mm 30° optic kit, 6703BA, Tuttlingen, Germany). Fentanyl (5 μg/bwkg; Richter Gedeon, Budapest, Hungary), dormicum (0.05 mg/bwkg; Egis Pharmaceuticals PLC, Budapest, Hungary), and ketamine (CP Ketamin 10% AUV; Medicus Partner, Hungary) were used as premedication before anesthesia induction using propofol (5.5 mg/bwkg; propofol 1% MCT/LCT; Fresenius Kabi AB, Bad Homburg, Germany). Anesthesia was maintained with a mixture of isoflurane and oxygen gas (Isoflutek 1,000 mg/g, 1.5% volume/volume; Laboratorios Karizoo SA, Barcelona, Spain). The buccal hair was removed, and the mouth area was disinfected using chlorhexidine (Curasept Chlorhex 30 mL, 0.5% spray, Curaden Swiss, Marleston, Australia). Subsequently, the soft palate was incised in the midline using an electrocautery device, and a hole was drilled into the basisphenoid bone according to the preoperatively determined landmarks. Under continuous endoscopic guidance, the hole was enlarged until the medial edges of both cavernous sinuses were visible around the pituitary gland. The cyst was drained and the entire pituitary gland was removed along with a part of the cyst wall. The opened third chamber was visible after complete gland removal. The bone defect was closed using a special bone reconstruction and anticoagulant sponge (Cerasorbe Foam; Curasan AG, Kleinostheim, Bayern, Bavaria, Germany), and the soft palate wound was closed with monofilament absorbable sutures (Surgicryl, SMI AG, Hünningen, Belgium).

During and 24 h after surgery, strict monitoring and tests were conducted at 1-h intervals. Examinations included measurement of the body temperature, blood pressure, acid-base status, electrolyte status, urine specific gravity and volume, heart and respiratory rates, blood lactate and glucose levels, and tear production. No abnormalities were noted.

Substitution therapy was initiated immediately after surgery. This included intramuscular hydrocortisone injections (Solu-Cortef 1 mg/bwkg TID; Pfizer, New York City, NY, USA) and desmopressin eye drops (Nocutil 0.1 mg/mL spray, one drop TID; Gebro Pharma GmbH, Fieberbrunn, Austria). A buprenorphine injection (Bupredine Multidose 0.3 mg/mL, 0.03 mg/bwkg; Produlab Pharma B.V., Raamsdonksveer, Netherlands) was administered every 6 h, while an amoxicillin-clavulanic acid injection (Augmentin 1000/200 mg, 20 mg/bwkg; GlaxoSmithKline Pharmaceuticals, Ltd., Brentford, UK) was intravenously administered BID for 3 days. The surgery and the first post-operative day were uneventful.

**Figure 2:**
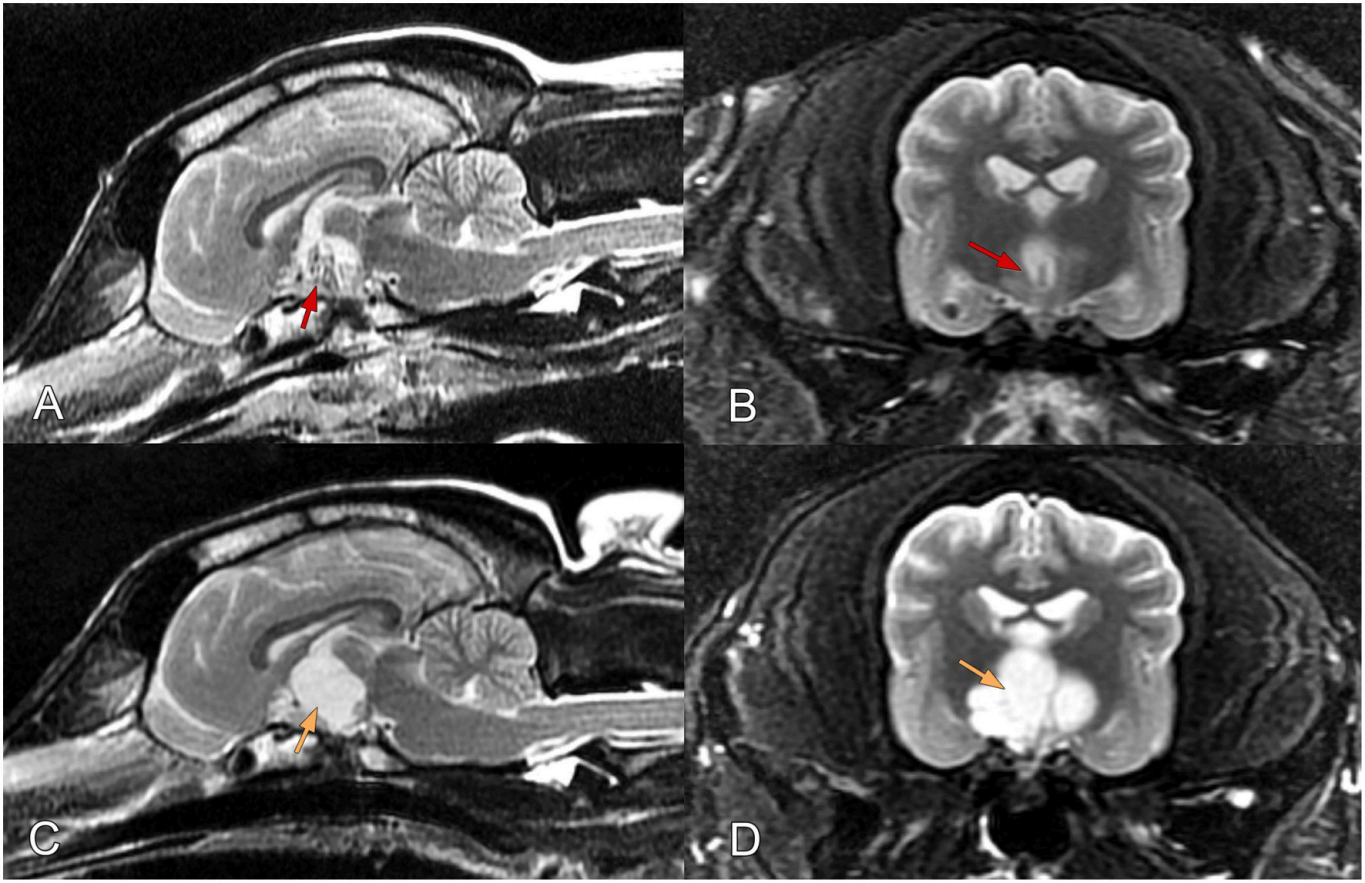
**(A,B)** Findings of post-operative magnetic resonance imaging of the brain in a female mixed-breed dog with a suprapituitary ependymal cyst managed via endoscopic transsphenoidal surgery. **(A)** Sagittal and **(B)** transverse T2-weighted images obtained 1 day after surgery. The images show removal of the hypophysis and complete drainage of the cyst (red arrows pointing to the empty cyst). **(C,D)** T2-weighted images obtained 73 days after surgery. Orange arrows pointing to the refilled cyst.

One day after surgery, MRI was performed using the same 1.5-T scanner (Siemens) to acquire the following sequences: T2W fast spin echo images in the transverse (TE/TR, 83.3/3,920 ms; SL, 3 mm) and sagittal (TE/TR, 96.5/4,500 ms; SL, 3 mm) planes; T2W fluid-attenuated inversion recovery images in the dorsal plane (TE/TR, 127.4/8,002 ms; SL, 3 mm); T2W gradient recalled echo images in the horizontal plane (TE/TR, 20/620); T1-weighted spin echo images in the sagittal plane (TE/TR, 15/240 ms; SL, 2 mm); post-contrast images (0.2 mL/bwkg; Dotarem 0.05 mmol/l injection, Guerbet, Villepinte, France); and three-dimensional T1W images (magnetization prepared rapid gradient-echo) in the sagittal, transverse, and dorsal planes. The sella turcica and cyst were both empty, although the cyst walls showed contrast enhancement on post-contrast images (Figures 2A,B).

Cytological analysis of the surgical specimen indicated a high probability of a neuroendocrine or ependymal origin of the mass.

The presence of normal adenohypophyseal tissue was observed on histopathological analysis of hematoxylin- and eosin-stained slides; intact acidophilic, basophilic, and chromophobe cell populations; acute hypophyseal hyperemia; and dilatation of the intrahypophyseal vessels filled with erythrocytes. Immunohistochemical examination of the intact adenohypophyseal cells showed diffuse, intense, homogeneous cytoplasmic synaptophysin-positivity; multifocal, intense, homogeneous cytoplasmic pancytokeratin-positivity; and Ki-67-negativity. The intact endothelial cells in the intrahypophyseal vessels showed diffuse, intense, homogeneous, and membranous CD31-positivity.

No abnormalities were observed on neurological examination performed on the first post-operative day, and 2 days after hospitalization, the dog regained vision in both eyes.

Normal tear production was observed on Schirmer's test. The dog was discharged 3 days after surgery. Oral administration of prednisolone (Prednisolon-Richter 5 mg, 1 mg/kg BID; POS Richter Gedeon), desmopressin eye drops (one drop TID; Gebro Pharma GmbH), oral amoxicillin-clavulanic acid (Synulox 250 mg BID; Zoetis, Parsippany-Troy Hills, NJ, USA), and oral levothyroxine sodium (L-thyroxin Henning 100 μg, 15 μg/bwkg BID, Sanofi Aventis, Paris, France) were continued at home.

Blood and urine tests were repeated at 1, 5, 10, 20, and 25 days after surgery. Moderate increase in alanine aminotransferase (mean, 315 IU/L), gamma glutamyltransferase (mean, 71.7 IU/L), lipase (mean, 1,871 IU/L), and alkaline phosphatase (man, 1,173 U/L) levels was observed in complete blood count, a chemistry panel, and urinalysis. The T4 level slightly decreased (8.7 nmol/l) in the initial post-operative period, although it normalized by day 25 (20.2 nmol/l).

However, the dog presented with the same preoperative signs and symptoms 73 days after surgery, exhibiting obtundation and depression, and a tendency of seeking tight spaces again. No other problems were observed on neurological examination, and normal findings were obtained on thoracic radiography and abdominal ultrasound. Regrowth of the cyst to its original size was apparent on MRI, and was compressing the surrounding structures (Figures 2C,D). Because of the poor prognosis and worsening clinical signs, the owner requested euthanasia.

**Figure 3:**
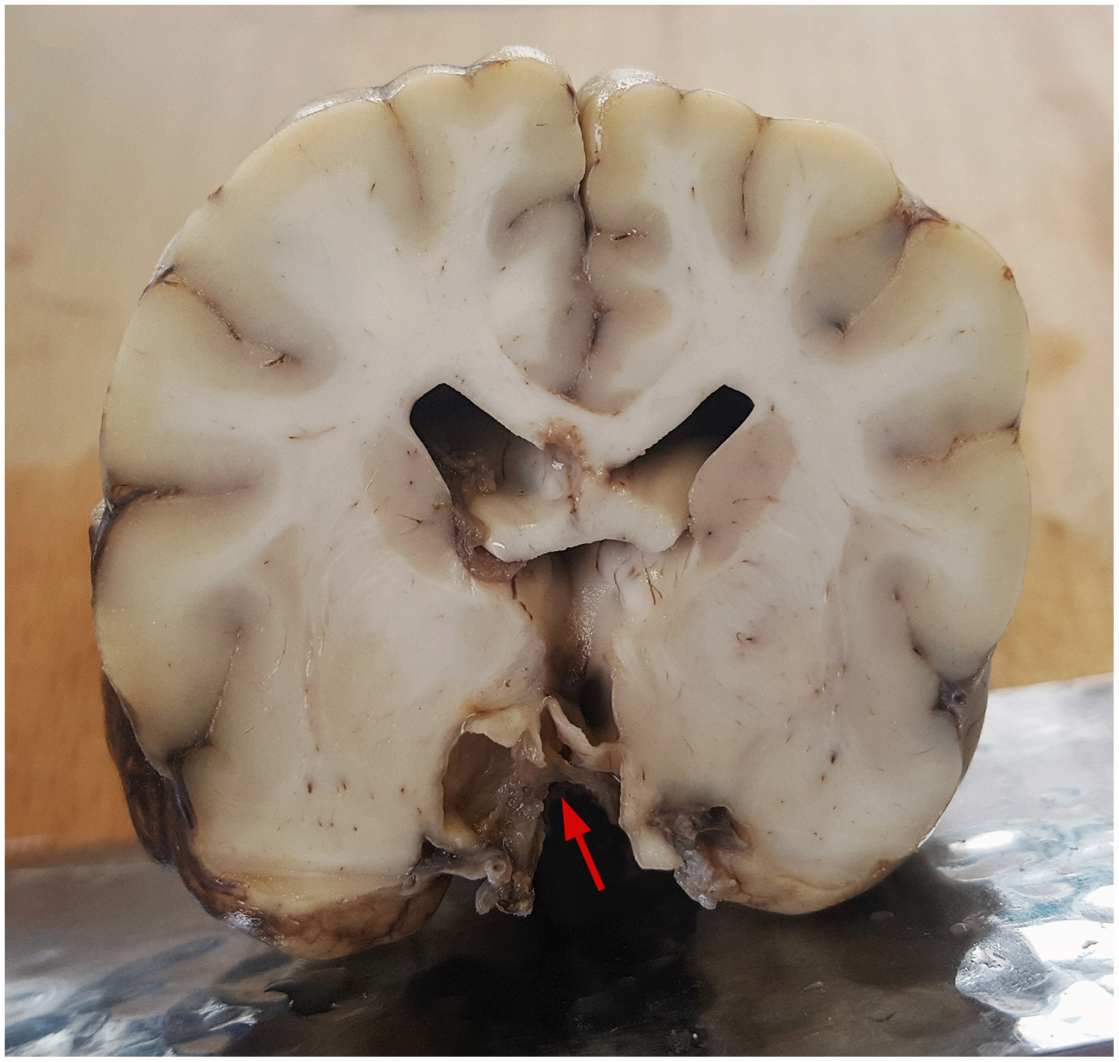
Histopathological analysis of a transverse section obtained during necropsy from the midthalamic region of the formalin-fixed brain of a female mixed-breed dog with a recurrent suprapituitary ependymal cyst. The arrow show the location of the cyst.

Macroscopically, the location of the cyst was accurately identified on the formalin-fixed brain *ex situ* (Figure 3). The wall of the intracerebral cyst consisted of a thin external layer of astrocytic glial tissue, as observed on histopathological analysis of hematoxylin- and eosin-stained slides prepared from the brain sample obtained during necropsy, and internal single and multifocally double layers of ciliated, cuboidal-to-flattened, ependyma-like cells. Positivity of the cyst wall for glial fibrillary acidic protein, S100 protein, and vimentin, and negativity for synaptophysin and epithelial membrane antigen was observed on immunohistochemical examination. Thus, the final diagnosis was ependymal cyst.

## Discussion

Intracranial cysts can compress the brain, and the neurological signs depend on the severity and localization of this compression. An enlarging cyst located above the pituitary gland can compress the interthalamic and thalamic regions and the optic chiasm, leading to dullness, depression, and blindness. In the present report, we describe a suprapituitary ependymal cyst that was treated by endoscopic transsphenoidal surgery in a mixed-breed dog. Current literature pertaining to veterinary medicine does not document any cases involving such lesions in dogs, although one study has described Rathke's cleft cyst (RCC) secreting antidiuretic hormone in a cat (31). In human medicine, RCCs are benign lesions that arise within the sella, between the anterior and posterior lobes of the pituitary gland, and can compress the surrounding structures of the sella turcica (optic chiasm and pituitary gland); RCCs are incidentally detected in 4–33% human autopsies (32). T2W and T1W images show heterogeneous signal intensity and hyperintensity, respectively. Sellar and parasellar cysts in humans are divided into four categories (33): pars intermedia cysts, RCCs, arachnoid cysts, and miscellaneous cysts. Pars intermedia cysts occasionally include colloid cysts, which are located between the anterior and posterior lobes of the pituitary gland, and do not communicate with the subarachnoid space. In human medicine, MRI can aid in differentiating pituitary cystic adenomas from RCCs (34).

Surgery is the only solution for effective reduction of the cyst size. The transsphenoidal technique (25–27, 35) used in this case was a logical approach because of the location of the pituitary gland and the related cyst, but it also restricted the manipulations due to the small exposure. In human medicine, hypophysectomy is primarily performed via an endoscopic endonasal approach, which was used for total cyst removal in 55.1% cases and subtotal resection in 44.83% cases in a human study (36). The cysts removed in their entirety did not recur after surgery, whereas subtotally resected cysts showed regrowth in 30.8% cases within 60 months after the surgery (36). As mentioned above, suprasellar cyst-like lesions have not been reported in veterinary medicine. In the present case, pituitary gland removal and partial ablation of the cyst wall eliminated the clinical signs and symptoms for a certain period of time. However, because the wall was not completely removed (due to its adherence to the adjacent brain tissues and because of the technological limitations during intracranial manipulation), the cyst eventually refilled, and resulted in recurrence of the clinical signs and symptoms. In human neurosurgery, the management of arachnoid cysts includes open craniotomy and cyst marsupialization followed by the insertion of a cystoperitoneal shunt (35, 37, 38). These methods cannot be used for pituitary gland surgery in dogs, in which the location of intracranial cysts can be very diverse. The most common sites of intracranial cysts in animals include the fourth ventricle and cerebellopontine angle (2, 6, 8, 10, 12, 14–16) and the area above the quadrigeminal plate (4–7, 9). There are individual reports of cysts located in the brainstem (6, 9, 11, 13); cerebellum (3); and frontal (11), diencephalic (1), and hypophyseal (31) regions. Lesions involving the cerebellar, medullary, and fourth ventricular regions can be best approached from the caudal direction (suboccipital approach, e.g., cystoperitoneal shunting). Frontal lesions affecting the olfactory bulb and frontal cerebral region can be accessed via a dorsal (transfrontal) approach; QCs can be approached dorsally or dorsolaterally (rostro- or caudotentorial approach), while diencephalic lesions (involving the thalamus, hypothalamus, or hypophysis) can be accessed via ventral (transsphenoidal) or dorsal (transfrontal or rostrotentorial), and occasionally, lateral (transtemporal) approaches (39, 40).

In the present case, we decided to use a ventral (transsphenoidal) approach for cyst drainage, combined with removal of the pituitary gland, for the following reasons. First, MRI strongly indicated that the intrasellar component of the lesion was etiologically associated with the pituitary gland. Second, the features of the lesion appeared similar to those of RCCs described in human (32–34) and veterinary (31) medicine. In addition, although we performed cytological analysis only after the surgery (which involved removal of the hypophysis), the findings did not exclude a neuroendocrine origin. We strongly believe that the cytological diagnosis would have led to the surgeon opting for a second surgical intervention for removal of the presumably tumorous pituitary gland if the cyst had been drained via a dorsal (transcallosal) approach.

To the best of our knowledge, this is the first report of an ependymal cyst with intra- and suprasellar involvement, and its surgical management in a canine. If involvement of the pituitary gland is confirmed or strongly suspected on the basis of cytological and/or imaging findings, drainage together with hypophysectomy via an endoscopic transsphenoidal approach could be the best surgical strategy. A dorsal approach should be considered only for fine needle aspiration and cyst drainage, because exposure and complete removal of the cystic lesion via this approach could result in severe damage to the surrounding brain tissues, particularly if the cyst is too large or located deep within the brain. However, complete removal of the cyst (together with its wall) using the endoscopic transsphenoidal approach is also very challenging because it provides limited access and allows visualization of only a small intracranial area. Similar future cases could help in better decision-making, such as assessing the direction of the surgical approach; draining off or continuous shunting the content of a cyst; and the need of concurrent hypophysectomy, depending on cytological analysis results.

## Ethics Statement

This study was carried out in accordance with regulations of the Hungarian Animal Health Care and guidelines of the veterinary science. As we report a case study of veterinary hospital we have the consent of both the owner and veterinarian that this dog (underwent the listed examinations and surgical intervention) was treated not for experimental but for medical reason. Due to the latter there was no need for approval of an ethical committee.

## Author Contributions

LL and KC: concept and original draft, collecting data, editing and reviewing draft; RG, CJ, and ZK: collecting data, reviewing draft.

### Conflict of Interest Statement

The authors declare that the research was conducted in the absence of any commercial or financial relationships that could be construed as a potential conflict of interest.
